# Clonal Hematopoiesis and Incident Heart Failure With Preserved Ejection Fraction

**DOI:** 10.1001/jamanetworkopen.2023.53244

**Published:** 2024-01-25

**Authors:** Art Schuermans, Michael C. Honigberg, Laura M. Raffield, Bing Yu, Mary B. Roberts, Charles Kooperberg, Pinkal Desai, April P. Carson, Amil M. Shah, Christie M. Ballantyne, Alexander G. Bick, Pradeep Natarajan, JoAnn E. Manson, Eric A. Whitsel, Charles B. Eaton, Alexander P. Reiner

**Affiliations:** 1 Program in Medical and Population Genetics and Cardiovascular Disease Initiative, Broad Institute of Harvard and MIT, Cambridge, Massachusetts; 2 Cardiovascular Research Center and Center for Genomic Medicine, Massachusetts General Hospital, Boston; 3 Faculty of Medicine, KU Leuven, Leuven, Belgium; 4 Department of Medicine, Harvard Medical School, Boston, Massachusetts; 5 Department of Genetics, University of North Carolina, Chapel Hill; 6 School of Public Health, The University of Texas Health Science Center, Houston; 7 Center for Primary Care and Prevention, Brown University, Pawtucket, Rhode Island; 8 Public Health Sciences Division, Fred Hutchinson Cancer Center, Seattle, Washington; 9 Division of Hematology and Oncology, Weill Cornell Medical College, New York, New York; 10 Department of Medicine, University of Mississippi Medical Center, Jackson; 11 Division of Cardiovascular Medicine, University of Texas Southwestern Medical Center, Dallas; 12 Department of Medicine, Baylor College of Medicine, Houston, Texas; 13 Division of Genomic Medicine, Department of Medicine, Vanderbilt University Medical Center, Nashville, Tennessee; 14 Division of Preventive Medicine, Brigham and Women’s Hospital, Harvard Medical School, Boston, Massachusetts; 15 Department of Epidemiology, Harvard T.H. Chan School of Public Health, Boston, Massachusetts; 16 Department of Epidemiology, Gillings School of Global Public Health and Department of Medicine, School of Medicine, University of North Carolina, Chapel Hill; 17 Department of Epidemiology, Brown University, Providence, Rhode Island; 18 Care New England, Center for Primary Care and Prevention, Pawtucket, Rhode Island; 19 Department of Family Medicine, Warren Alpert Medical School of Brown University, Providence, Rhode Island

## Abstract

**Importance:**

Clonal hematopoiesis of indeterminate potential (CHIP), the age-related clonal expansion of hematopoietic stem cells with leukemogenic acquired genetic variants, is associated with incident heart failure (HF).

**Objective:**

To evaluate the associations of CHIP and key gene-specific CHIP subtypes with incident HF with preserved ejection fraction (HFpEF) and reduced ejection fraction (HFrEF).

**Design, Setting, and Participants:**

This population-based cohort study included participants from 2 racially diverse prospective cohort studies with uniform HF subtype adjudication: the Jackson Heart Study (JHS) and Women’s Health Initiative (WHI). JHS participants were enrolled during 2000 to 2004 and followed up through 2016. WHI participants were enrolled during 1993 to 1998 and followed up through 2022. Participants who underwent whole-genome sequencing, lacked prevalent HF at baseline, and were followed up for HF adjudication were included. Follow-up occurred over a median (IQR) of 12.0 (11.0-12.0) years in the JHS and 15.3 (9.0-22.0) years in the WHI. Statistical analysis was performed from June to December 2023.

**Exposures:**

Any CHIP and the most common gene-specific CHIP subtypes (*DNMT3A* and *TET2* CHIP).

**Main Outcomes and Measures:**

First incident hospitalized HF events were adjudicated from hospital records and classified as HFpEF (left ventricular ejection fraction ≥50%) or HFrEF (ejection fraction <50%).

**Results:**

A total of 8090 participants were included; 2927 from the JHS (median [IQR] age, 56 [46-65] years; 1846 [63.1%] female; 2927 [100.0%] Black or African American) and 5163 from the WHI (median [IQR] age, 67 [62-72] years; 5163 [100.0%] female; 29 [0.6%] American Indian or Alaska Native, 37 [0.7%] Asian or Pacific Islander, 1383 [26.8%] Black or African American, 293 [5.7%] Hispanic or Latinx, 3407 [66.0%] non-Hispanic White, and 14 [0.3%] with other race and ethnicity). The multivariable-adjusted hazard ratio (HR) for composite CHIP and HFpEF was 1.28 (95% CI, 0.93-1.76; *P* = .13), and for CHIP and HFrEF it was 0.79 (95% CI, 0.49-1.25; *P* = .31). *TET2* CHIP was associated with HFpEF in both cohorts (meta-analyzed HR, 2.35 [95% CI, 1.34 to 4.11]; *P* = .003) independent of cardiovascular risk factors and coronary artery disease. Analyses stratified by C-reactive protein (CRP) in the WHI found an increased risk of incident HFpEF in individuals with CHIP and CRP greater than or equal to 2 mg/L (HR, 1.94 [95% CI, 1.20-3.15]; *P* = .007), but not in those with CHIP and CRP less than 2 mg/L or those with CRP greater than or equal to 2 mg/L without CHIP, when compared with participants without CHIP and CRP less than 2 mg/L.

**Conclusions and Relevance:**

In this cohort study, *TET2* CHIP was an independent risk factor associated with incident HFpEF. This finding may have implications for the prevention and management of HFpEF, including development of targeted therapies.

## Key Points

**Question:**

Are clonal hematopoiesis of indeterminate potential (CHIP) or certain CHIP driver genes associated with a specific heart failure (HF) subtype?

**Findings:**

In this cohort study of 2 racially diverse cohorts collectively including 8090 participants, *TET2* CHIP was independently associated with a 2.4-fold higher risk of incident HF with preserved ejection fraction. By contrast, there were no significant associations of CHIP with incident HF with reduced ejection fraction.

**Meaning:**

These results suggest that *TET2* CHIP is a risk factor associated with incident HF with preserved ejection fraction.

## Teaser

This cohort study evaluates the potential associations of clonal hematopoiesis of indeterminate potential (CHIP) and key gene-specific CHIP subtypes with incident heart failure with preserved and reduced ejection fraction.

## Introduction

Clonal hematopoiesis of indeterminate potential (CHIP), the clonal expansion of blood stem cells with preleukemic acquired genetic variants, is an age-related condition affecting approximately 10% of individuals aged at least 70 years.^1^ CHIP is most commonly associated with variants in genes involved in epigenetic regulation (*DNMT3A* and *TET2*) and portends an increased risk of hematologic malignant neoplasms and atherosclerotic cardiovascular disease.^1,2^ Recent work suggests CHIP is also an independent risk factor for incident heart failure (HF),^3^ with experimental models indicating a role for CHIP, especially *TET2* CHIP, in the development of cardiac dysfunction through inflammatory dysregulation and fibrotic remodeling.^4,5,6^ In observational studies, associations of CHIP with HF were found to be independent of coronary artery disease (CAD),^3^ implying CHIP may be involved in HF pathophysiology beyond simply reflecting the association between CHIP and atherosclerosis. Evaluating a potentially differential role of CHIP variants in HF with preserved ejection fraction (HFpEF) vs HF with reduced ejection fraction (HFrEF) may provide insights into aging-related disease mechanisms and inform development of prevention and treatment strategies.

## Methods

### Study Population

We tested the associations of CHIP and gene-specific CHIP subtypes with HFpEF and HFrEF in 2 cohorts with uniform HF subtype adjudication and data on potential confounders: the Jackson Heart Study (JHS) and Women’s Health Initiative (WHI) (eMethods in Supplement 1). The JHS is a prospective cohort including 5306 self-identified Black adults from Jackson, Mississippi, who enrolled during 2000 to 2004. Follow-up for incident HF occurred through adjudication starting in 2005, as described previously.^7^ The WHI is a prospective US study including 161 808 postmenopausal women. WHI participants enrolled during 1993 to 1998 in an observational study or clinical trial(s) of hormone therapy, calcium and vitamin D supplementation, or dietary modification. A subset of participants (n = 44 174) were followed for HF adjudication by yearly medical record abstraction of self-reported hospitalizations.^8^ We considered 3404 JHS participants and 11 010 WHI participants with whole-genome sequencing (WGS) through the National Heart, Lung and Blood Institute Trans-Omics for Precision Medicine (TOPMed) program for inclusion. We excluded participants with HF at blood draw or no HF adjudication and those with missing key covariates (eFigure in Supplement 1). Because previous work suggests that CHIP prevalence may differ across races and ethnicities,^1^ the present study incorporated race and ethnicity as covariates in statistical models. Race and ethnicity data were collected by self-report in both the JHS and WHI.

The Massachusetts General Brigham institutional review board approved these secondary data analyses. All participants included in the JHS and WHI provided written informed consent for study participation. This study followed the Strengthening the Reporting of Observational Studies in Epidemiology (STROBE) reporting guideline for cohort studies.

### Ascertainment of Exposures and Outcomes

CHIP was ascertained in both cohorts using WGS from blood-derived DNA.^9^ Somatic variants were called in 74 leukemia-associated genes (eTable 1 in Supplement 1), as described previously.^2,9^ In brief, putative somatic variants (including single-nucleotide polymorphisms and indels) were identified using GATK Mutect2.^10^ Recurrent sequencing artifacts were excluded using an external reference panel of normal samples including 100 randomly selected individuals under the age of 40 years.^9^ Likely germline variants were also excluded using an external reference of germline variants.^11^ Samples were classified as CHIP if Mutect2 detected at least 1 putative CHIP variant (eTable 1 in Supplement 1) at a variant allele frequency greater than 2%. Additional details on the TOPMed CHIP calling approach have been described previously.^9^ Given previous evidence suggesting important heterogeneity between gene-specific CHIP subtypes,^12,13,14^ we examined the most common gene-specific CHIP subtypes (*DNMT3A* and *TET2*) as the coprimary study exposures. Any CHIP, as well as large (variant allele fraction [VAF] >10%) CHIP, *DNMT3A* CHIP, and *TET2* CHIP, were tested as secondary exposures because previous work suggests that larger clones are more strongly associated with some cardiovascular outcomes.^3,13,15^

HF adjudication procedures during follow-up were uniform across the JHS and WHI.^7,8^ HF events were classified as HFpEF (left ventricular ejection fraction ≥50%) or HFrEF (ejection fraction <50%) based on imaging data before and/or at the time of HF hospitalization. Adjudicated HF events without data on ejection fraction were classified as HF with unknown ejection fraction (HFuEF). Time to HFpEF and time to HFrEF were the coprimary study outcomes.

### Statistical Analysis

Baseline participant characteristics were presented as median (IQR) or number and frequency; and they were compared between those with vs without CHIP using the Wilcoxon rank-sum test for continuous variables and the Pearson χ^2^ or Fisher exact test for categorical variables as appropriate. We used the Kaplan-Meier method and log-rank test to compare the cumulative incidence of any HF, HFpEF, and HFrEF by CHIP carrier status. Associations of CHIP and gene-specific CHIP subtypes with incident events were tested using Cox proportional hazards models adjusted for age, income, education, smoking, body mass index, systolic blood pressure, antihypertensive medication, prevalent diabetes, CAD, and stroke. JHS models were further adjusted for sex, and WHI models were further adjusted for self-reported race and ethnicity. All multivariable-adjusted WHI analyses were also weighted by inverse sampling probabilities to allow inferences to the full WHI cohort from which the TOPMed subsample (ie, a case-control sample enriched for stroke and venous thromboembolism) and the HF adjudication participants were drawn.^16^ Inverse sampling probability weights accounting for selection patterns in the WHI were calculated using multivariable-adjusted logistic regression models including age, body mass index, race and ethnicity, WHI substudy (observational study vs clinical trial), hysterectomy status, deep venous thromboembolism, pulmonary embolism, stroke, hemorrhagic stroke, and ischemic stroke.

Participants who died without experiencing an HF event were censored as nonevents at their time of death; others who did not experience an HF event were censored as nonevents at the end of follow-up. For each HF subtype (eg, HFpEF), participants with other HF subtypes as their first event (eg, HFrEF or HFuEF) were censored as nonevents. WHI follow-up was truncated at 22 years due to deviations from proportional hazards. The proportional hazards assumption was verified by plotting Schoenfeld residuals against time; the linearity assumption was verified by plotting Martingale residuals against the continuous covariates included in multivariable-adjusted Cox regression models. Because prior evidence suggests CHIP is associated with incident CAD and diabetes,^2,17^ sensitivity analyses tested the associations of CHIP and gene-specific CHIP subtypes with HF subtypes using models incorporating CAD and diabetes as time-varying covariates. Additional sensitivity analyses excluded all participants with CAD at baseline and stratified by age younger than 65 years vs 65 years or older.

Effect estimates were meta-analyzed with fixed-effects models using the rmeta^18^ (version 3.0) and meta^19^ (version 6.0-0) packages in R. The Cochran *Q* test and *I*^2^ statistic were used to test between-study heterogeneity. Two-tailed *P* < .0125 (.05 / 4) indicated statistical significance given 2 gene-specific CHIP subtypes (*DNMT3A* and *TET2*) and 2 outcomes (time to HFpEF and time to HFrEF) tested in primary analyses (multivariable-adjusted Cox regression analyses). Analyses were performed using R version 4.1.0 (R Project for Statistical Computing) from June to December 2023.

## Results

The study sample included 8090 participants, including 2927 from the JHS (median [IQR] age, 56 [46-65] years; 1846 [63.1%] female; 2927 [100.0%] Black or African American) and 5163 from the WHI (median [IQR] age, 67 [62-72] years; 5163 [100.0%] female; 29 [0.6%] American Indian or Alaska Native, 37 [0.7%] Asian or Pacific Islander, 1383 [26.8%] Black or African American, 293 [5.7%] Hispanic or Latinx, 3407 [66.0%] non-Hispanic White, and 14 [0.3%] with other race and ethnicity). A total of 512 participants had CHIP (JHS: 112 of 2927 [3.8%]; WHI: 400 of 5163 [7.7%]). Among CHIP carriers, 313 (61.1%) had driver variants in *DNMT3A* and 108 (21.1%) in *TET2*; the median (IQR) variant allele frequency was 15.5% (11.9%-22.9%). Baseline participant characteristics are listed in Table 1. JHS participants were followed up for a median (IQR) of 12.0 (11.0-12.0) years, and WHI participants were followed up for a median (IQR) of 15.3 (9.0-22.0) years. There were 251 incident HF cases (120 HFpEF; 102 HFrEF) in the JHS and 694 (339 HFpEF; 237 HFrEF) in the WHI.

**Table 1.** Characteristics of Participants Included in the Jackson Heart Study and Women’s Health Initiative by CHIP Carrier Status at Time of Blood Draw

Characteristic	Jackson Heart Study			Women’s Health Initiative		
	Participants without CHIP (n = 2815)	Participants with CHIP (n = 112)	*P* value^a^	Participants without CHIP (n = 4763)	Participants with CHIP (n = 400)	*P* value^a^
Age at blood draw, median (IQR), y^b^	55 (45-64)	65 (60-74)	<.001	67 (62-72)	69 (65-74)	<.001
Sex, No. (%)^b^						
Female	1778 (63.2)	68 (60.7)	.67	4763 (100.0)	400 (100.0)	NA
Male	1037 (36.8)	44 (39.3)		0	0	
Race and ethnicity, No. (%)^b^						
American Indian or Alaska Native	0	0	NA	24 (0.5)	5 (1.3)	.06
Asian or Pacific Islander	0	0		35 (0.7)	2 (0.5)	
Black or African American	2815 (100.0)	112 (100.0)		1292 (27.1)	91 (22.8)	
Hispanic or Latinx	0	0		276 (5.8)	17 (4.3)	
Non-Hispanic White	0	0		3122 (65.5)	285 (71.2)	
Other^c^	0	0		14 (0.3)	0	
Education, No. (%)^b^						
No high school degree	435 (15.5)	28 (25.0)	.08	399 (8.4)	23 (5.8)	.49
High school	566 (20.1)	21 (18.8)		926 (19.4)	79 (19.8)	
Some college/trade school^d^	1806 (64.2)	63 (56.2)		1864 (39.1)	163 (40.8)	
College or higher	NA	NA		1448 (30.4)	124 (31.0)	
Missing	8 (0.3)	0		126 (2.6)	11 (2.8)	
Income class, No. (%)^b^^,^^e^						
Lower	294 (10.4)	14 (12.5)	.55	579 (12.2)	48 (12.0)	.38
Lower-middle	569 (20.2)	23 (20.5%)		1276 (26.8)	113 (28.2)	
Upper-middle	758 (26.9)	23 (20.5)		914 (19.2)	91 (22.8)	
Affluent	770 (27.4)	31 (27.7)		693 (14.5)	51 (12.8)	
Very affluent	NA	NA		464 (9.7)	31 (7.8)	
Missing	424 (15.1)	21 (18.8)		837 (17.6)	66 (16.5)	
Smoking, No. (%)^b^						
Current	346 (12.3)	11 (9.82)	.08	470 (9.9)	26 (6.5)	.03
Past	549 (19.5)	33 (29.5)		1791 (37.6)	169 (42.2)	
Never	1916 (68.1)	68 (60.7)		2429 (51.0)	203 (50.7)	
Missing	4 (0.1)	0		73 (1.5)	2 (0.5)	
BMI ^b^	30.6 (26.9-35.5)	30.1 (26.3-34.0)	.27	28.8 (25.2-33.2)	29.0 (25.5-32.4)	.95
Biochemistry, median (IQR), mg/dL^f^						
Total cholesterol	196 (173-223)	198 (170-229)	.68	222 (197-248)	221 (195-247)	.22
Low-density lipoprotein cholesterol	125 (101-148)	120 (103-151)	.89	138 (115-163)	137 (110-163)	.19
High-density lipoprotein cholesterol	50 (41-60)	51.0 (40-61)	.67	54 (45-64	53 (45-63)	.39
Triglycerides	90 (64-127)	87 (63-132)	.85	128 (94-177)	132 (97-172)	.49
Systolic blood pressure, median (IQR), mm Hg^b^	126 (116-136)	133 (121-146)	<.001	130 (119-142)	130 (120-141)	.46
Antihypertensive medication, No. (%)^b^	1423 (50.6)	82 (73.2)	<.001	1569 (32.9)	125 (31.2)	.52
Prevalent diabetes at blood draw, No. (%)^b^	635 (22.6)	33 (29.5)	.11	529 (11.1)	30 (7.5)	.03
Prevalent coronary artery disease at blood draw, No. (%)^b^	95 (3.4)	6 (5.4)	.28	207 (4.3)	15 (3.8)	.66
Prevalent stroke at blood draw, No. (%)^b^	100 (3.6)	3 (2.7)	.80	135 (2.8)	10 (2.5)	.82
Left ventricular ejection fraction at study enrollment, %^g^	65 (55-65)	65 (55-65)	.14	NA	NA	NA

Abbreviations: BMI, body mass index (calculated as weight in kilograms divided by height in meters squared); CHIP, clonal hematopoiesis of indeterminate potential; NA, not applicable.

^a^ Participant characteristics were compared between those with vs without CHIP using the Wilcoxon rank-sum test for continuous variables and the Pearson χ^2^ or Fisher exact test for categorical variables as appropriate.

^b^ There were no missing data for these variables, or missing was considered as a separate category.

^c^ The other race and ethnicity category does not include any particular predefined races and ethnicities; WHI participants were included in this category if they did not identify as American Indian or Alaskan Native, Asian or Pacific Islander, Black or African American, Hispanic or Latinx, or White (not of Hispanic origin).

^d^ The JHS made no distinction between some college or trade school and college or higher.

^e^ In the JHS, income categories were defined by household income less than 1 (lower), 1 to 1.5 (lower-middle), 1.5 to 3.5 (upper-middle), and greater than or equal to 3.5 (affluent) times the poverty level. In the WHI, income categories were defined by household income less than $20 000 (lower), $20 000 to $34 999 (lower-middle), $35 000 to $49 999 (upper-middle), $50 000 to $74 999 (affluent), or greater than $75 000 (very affluent).

^f^ Total cholesterol was missing for 201 JHS participants and 497 WHI participants; low-density lipoprotein cholesterol was missing for 226 JHS participants and 546 WHI participants, high-density lipoprotein cholesterol was missing for 201 JHS participants and 498 WHI participants, and triglycerides was missing for 201 JHS participants and 504 WHI participants.

^g^ Left ventricular ejection fraction was missing for 107 JHS participants.

**Figure 1.**
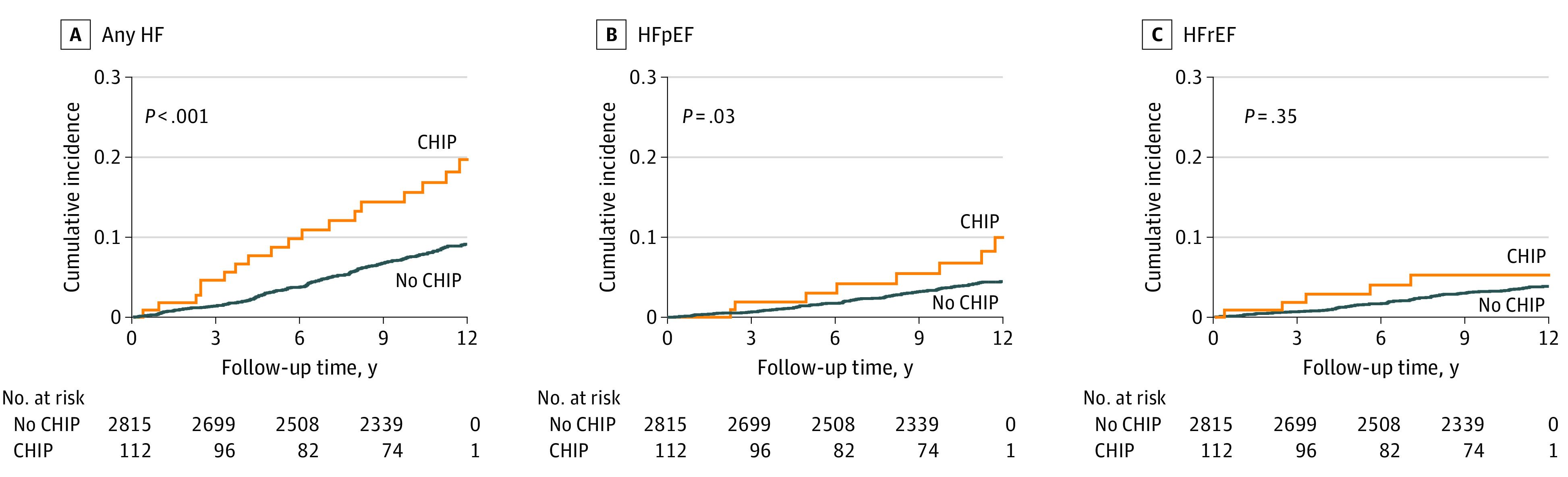
Cumulative Incidence of Heart Failure (HF) and HF Subtypes by Clonal Hematopoiesis of Indeterminate Potential (CHIP) Carrier Status in the Jackson Heart Study (JHS) Cumulative incidence curves for any HF, HF with preserved ejection fraction (HFpEF), and HF with reduced ejection fraction (HFrEF) were constructed using the Kaplan-Meier method and compared using the (unadjusted) log-rank test. Any HF was defined as a composite outcome including HFpEF, HFrEF, and HF with unknown ejection fraction. Follow-up occurred over a median (IQR) of 12.0 (11.0-12.0) years.

**Figure 2.**
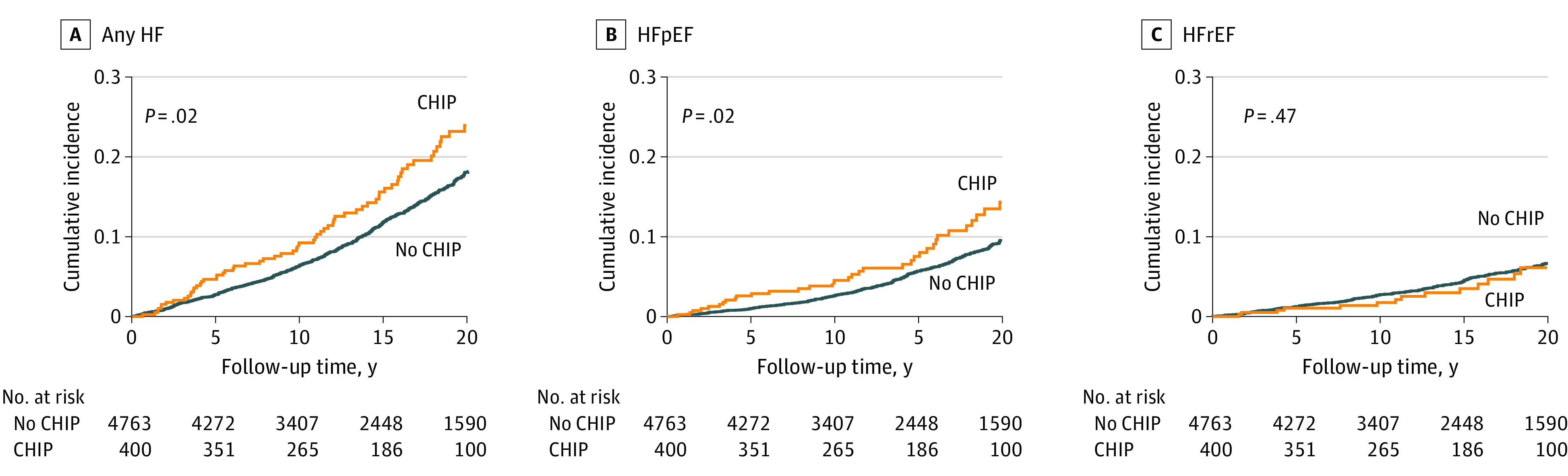
Cumulative Incidence of Heart Failure (HF) and HF Subtypes by Clonal Hematopoiesis of Indeterminate Potential (CHIP) Carrier Status in the Women’s Health Initiative (WHI) Cumulative incidence curves for any HF, HF with preserved ejection fraction (HFpEF), and HF with reduced ejection fraction (HFrEF) were constructed using the Kaplan-Meier method and compared using the (unadjusted) log-rank test. Any HF was defined as a composite outcome including HFpEF, HFrEF, and HF with unknown ejection fraction. Follow-up was truncated at 22 years due to deviations from proportional hazards and occurred over a median (IQR) of 15.3 (9.0-22.0) years.

The cumulative incidence of any HF and HFpEF, but not HFrEF, was higher among CHIP carriers vs noncarriers in the JHS (Figure 1) and WHI (Figure 2). While multivariable-adjusted analyses did not reveal statistically significant associations of any CHIP with HFpEF (meta-analyzed HR, 1.28 [95% CI, 0.93-1.76]; *P* = .13) or HFrEF (meta-analyzed HR, 0.79 [95% CI, 0.49-1.25]; *P* = .31), *TET2* CHIP was significantly associated with HFpEF (meta-analyzed HR, 2.35 [95% CI, 1.34-4.11]; *P* = .003) (Table 2). *DNMT3A* CHIP was not significantly associated with any HF, HFrEF, or HFpEF. Results were consistent in sensitivity analyses incorporating diabetes and CAD as time-varying covariates (eTable 2 in Supplement 1) and in those excluding participants with prevalent CAD (eTable 3 in Supplement 1). There were no statistically significant differences between participants aged younger than 65 years vs 65 years or older for the associations of CHIP and gene-specific CHIP subtypes with HFpEF and HFrEF (eTables 4, 5, and 6 in Supplement 1), although absolute case counts in the group aged younger than 65 years were low. Secondary analyses testing large CHIP clones (ie, CHIP with VAF >10%) found materially unchanged associations for *TET2* across HF subtypes (eTable 7 in Supplement 1).

**Table 2.** Multivariable-Adjusted Associations of CHIP and Gene-Specific CHIP Subtypes With Incident HFpEF and HFrEF

Outcome and exposure	Jackson Heart Study			Women’s Health Initiative			Meta-analysis			
	Cases/total No. (%)	HR (95% CI)^a^	*P* value	Cases/total No. (%)	HR (95% CI)^a^	*P* value	HR (95% CI)^b^	*P* value	*P* value for between-study heterogeneity (Cochran *Q*)	*I*^2^ statistic (%)
Any HF										
Any CHIP	18/112 (16.1)	1.19 (0.73-1.94)	.48	65/400 (16.3)	1.23 (0.95-1.59)	.12	1.22 (0.97-1.53)	.09	.92	0.0
*DNMT3A* CHIP	12/68 (17.6)	1.27 (0.70-2.31)	.42	34/245 (13.8)	1.02 (0.72-1.44)	.91	1.08 (0.80-1.46)	.62	.53	0.0
*TET2* CHIP	6/21 (28.6)	3.3 (1.44-7.54)	.005	20/87 (23.0)	1.93 (1.23-3.02)	.004	2.18 (1.47-3.23)	<.001	.26	20.5
HFpEF										
Any CHIP	8/112 (7.1)	1.09 (0.53-2.25)	.82	35/400 (8.8)	1.33 (0.94-1.90)	.11	1.28 (0.93-1.76)	.13	.62	0.0
*DNMT3A* CHIP	4/68 (5.9)	0.85 (0.31-2.35)	.76	20/245 (8.2)	1.20 (0.76-1.89)	.44	1.13 (0.74-1.71)	.57	.55	0.0
*TET2* CHIP	3/21 (14.3)	3.97 (1.22-12.92)	.02	10/87 (11.5)	2.01 (1.07-3.81)	.03	2.35 (1.34-4.11)	.003	.32	0.0
HFrEF										
Any CHIP	5/112 (4.5)	0.81 (0.32-2.01)	.65	14/400 (3.5)	0.78 (0.45-1.34)	.37	0.79 (0.49-1.25)	.31	.95	0.0
*DNMT3A* CHIP	3/68 (4.4)	0.78 (0.24-2.51)	.68	4/245 (1.6)	0.36 (0.13-0.97)	.04	0.50 (0.23-1.06)	.07	.32	0.0
*TET2* CHIP	2/21 (9.5)	2.34 (0.57-9.66)	.24	5/87 (5.7)	1.40 (0.58-3.43)	.46	1.62 (0.76-3.45)	.21	.55	0.0

Abbreviations: CHIP, clonal hematopoiesis of indeterminate potential; HF, heart failure; HFpEF, heart failure with preserved ejection fraction; HFrEF, heart failure with reduced ejection fraction; HR, hazard ratio.

^a^ HRs with corresponding 95% CIs were calculated using Cox proportional hazards models adjusted for age at blood draw, income, education, smoking, body mass index, systolic blood draw, antihypertensive medication use, prevalent diabetes, coronary artery disease, and stroke. JHS models were further adjusted for sex, and WHI models were further adjusted for race and ethnicity and inverse probability weights for inclusion in the Trans-Omics for Precision Medicine and HF adjudication cohorts. Any HF was defined as a composite outcome including HFpEF, HFrEF, and HF with unknown ejection fraction. Follow-up in the WHI was truncated at 22 years due to deviations from proportional hazards. Follow-up occurred over a median (IQR) of 12.0 (11.0-12.0) years in the JHS and 15.3 (9.0-22.0) years in the WHI.

^b^ Effect estimates from both cohorts were meta-analyzed using fixed effects.

**Figure 3.**
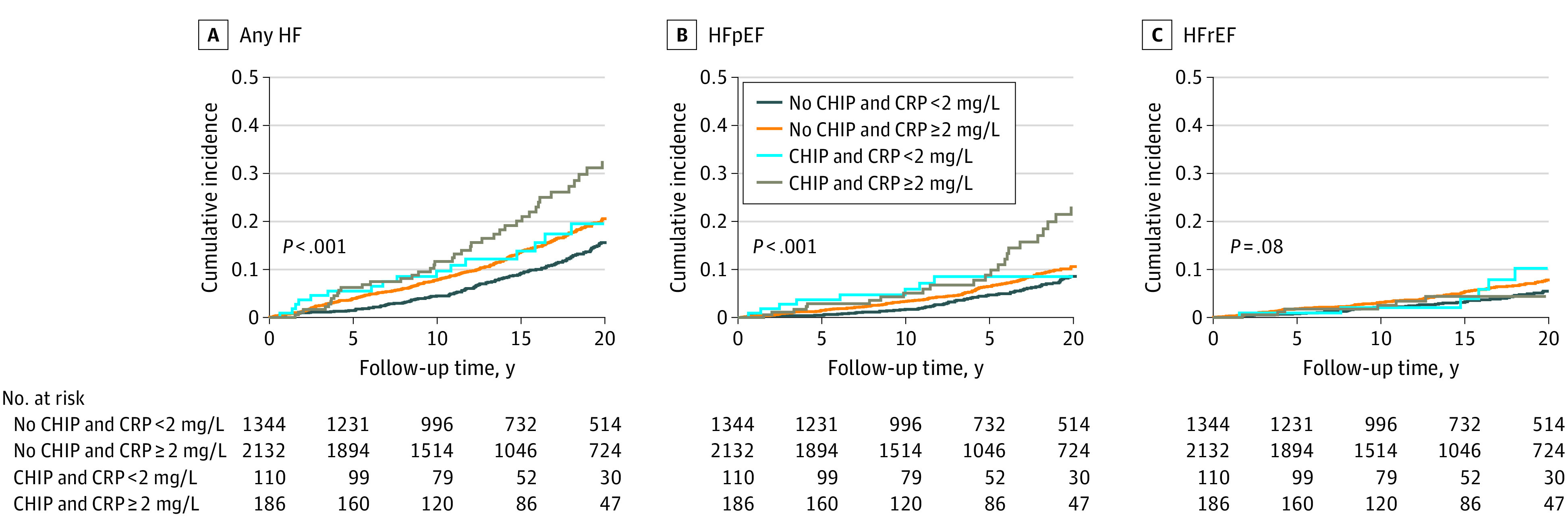
Cumulative Incidence of Heart Failure (HF) and HF Subtypes by Clonal Hematopoiesis of Indeterminate Potential (CHIP) Carrier Status and C-Reactive Protein (CRP) Levels in the Women’s Health Initiative (WHI) Cumulative incidence curves for any HF, HF with preserved ejection fraction (HFpEF), and HF with reduced ejection fraction (HFrEF) were constructed using the Kaplan-Meier method and compared using the log-rank test. Any HF was defined as a composite outcome including HFpEF, HFrEF, and HF with unknown ejection fraction. A total of 3643 WHI participants had available CRP measurements. Follow-up was truncated at 22 years due to deviations from proportional hazards and occurred over a median (IQR) of 15.3 (9.0-22.0) years.

Given experimental evidence implicating inflammation in the association of CHIP with cardiac dysfunction,^4,5,6^ we tested whether the associations of CHIP with incident HF subtypes differed according to baseline C-reactive protein (CRP) levels in the WHI. The cumulative incidence of any HF and HFpEF were higher in CHIP carriers with CRP greater than or equal to 2 mg/L compared with CHIP carriers with CRP less than 2 mg/L (Figure 3). Similar differences were not observed for HFrEF. Although no statistically significant interaction was detected, multivariable-adjusted analyses found that, compared with participants with no CHIP and CRP less than 2 mg/L, CHIP with CRP greater than or equal to 2 mg/L was associated with a 1.94-fold higher HFpEF risk (95% CI, 1.20-3.15; *P* = .007), whereas neither CHIP with CRP less than 2 mg/L nor CRP greater than or equal to 2 without CHIP were significantly associated with HFpEF (eTable 8 in Supplement 1).

## Discussion

In 2 large, racially diverse cohorts, *TET2* CHIP was associated with a more than 2-fold higher risk of HFpEF, independent of traditional risk factors and CAD. CHIP carriers with CRP greater than or equal to 2 mg/L were at highest risk for incident HFpEF. Overall, our findings suggest that previously described associations between CHIP and incident HF may be driven primarily by HFpEF and indicate important differences among common CHIP subtypes. In particular, *TET2* CHIP represents a novel risk factor for HFpEF.

Although inflammation represents a common mechanism involved in both HFpEF and HFrEF, chronic inflammation and fibrotic remodeling driven by myeloid cells is increasingly recognized as a primary contributor to HFpEF.^20^ Animal models of CHIP, particularly *TET2* CHIP, support a role for CHIP-induced inflammatory dysregulation in the pathogenesis of cardiac remodeling, fibrosis, and ventricular dysfunction.^4,5,6^ Post-hoc analyses from the CANTOS trial^21^ suggested that individuals with *TET2* CHIP may derive particular benefit from interleukin-1β inhibition to reduce risk of CAD. The present study extends these findings by finding that CRP stratified the association of CHIP with HFpEF in the WHI, further implicating inflammation in the association of CHIP with cardiovascular diseases beyond CAD. Future studies should test whether CHIP, especially *TET2* CHIP, represents a potential target for modulation toward HFpEF prevention and treatment, potentially through inhibition of inflammatory pathways.

Although previous analyses of large-scale observational cohorts (including the JHS and WHI) have shown that CHIP may be associated with incident HF,^3^ studies on the potential role of CHIP in incident HFpEF and HFrEF have been limited. In a recent study including 358 individuals with incident HF and 347 age- and sex-matched control participants, CHIP was associated with higher natriuretic peptide levels, with subgroup analyses suggesting a nominally increased risk of incident HFpEF but not HFrEF among individuals younger than 65 years with vs without CHIP.^22^ Similarly, in a case-control study of 81 individuals with HFpEF vs 36 controls, Cochran et al^23^ found that those with HFpEF were significantly enriched for *TET2* CHIP. The same study found that mice modeled for *TET2* CHIP had increased diastolic dysfunction, ventricular hypertrophy, and cardiac fibrosis in comparison with control mice.^23^ Our results corroborate and extend these findings by showing that *TET2* CHIP was associated with incident HFpEF in a population-based sample, identifying *TET2* CHIP as a strong and independent risk factor for new-onset HFpEF. Additional work using targeted sequencing is needed to determine an optimal VAF threshold for the prognostic significance of *TET2*-driven clonal hematopoiesis in HFpEF and to define pragmatic approaches for incorporating CHIP as a clinical risk factor for HFpEF.

### Limitations

Although this study benefited from next-generation sequencing and rigorous adjudication of HF events, analyses must be interpreted within the context of this study’s limitations. First, this study had reduced power to detect gene-specific associations with HFrEF and examine other less common driver variants. While previous work suggests that CHIP driven by genes such as *ASXL1* or *JAK2* is strongly associated with incident HF,^3^ the limited number of individuals with CHIP driven by these genes precluded the evaluation of these genes’ associations with incident HFpEF and HFrEF. Second, although HF events were adjudicated by an expert review panel, HF subtype definitions relied solely on left ventricular ejection fraction. Although this approach aligns with most large-scale epidemiological studies of HFpEF and HFrEF,^24^ it may not fully account for the complexity of HF subtypes beyond left ventricular ejection fraction.^24^ Third, while this study benefited from the inclusion of racially diverse cohorts, certain demographic groups were still not represented. However, recent case-control studies that mainly involved participants with demographics different from those in the present study (eg, White male participants) found consistent associations between CHIP or *TET2* CHIP and HFpEF,^22,23^ suggesting that our findings may generalize to other demographic groups. Fourth, while WGS enables large-scale genetic analyses in population-based cohorts, it has limited sensitivity to detect CHIP with VAF less than 10%.^9^ WGS may therefore misclassify a proportion of individuals with small CHIP clones; however, any such misclassification is expected to reduce the observed differences between those with vs without CHIP and result in bias toward the null. Despite the limited sensitivity of WGS to detect small CHIP clones, WGS can robustly identify CHIP carriers with CHIP with VAF greater than 10%.^9^ Although our findings are consistent with recent studies showing associations of CHIP or *TET2* CHIP with HFpEF using more sensitive approaches (such as those involving deep targeted sequencing),^22,23^ future studies are needed to clarify the effects of smaller CHIP clones on long-term risk of HFpEF and HFrEF.

## Conclusions

In this cohort study of 8090 participants, *TET2* CHIP was a risk factor associated with HFpEF, independent of traditional cardiovascular risk factors and CAD. Previously reported associations of CHIP with new-onset HF may primarily reflect associations with HFpEF. Further research into the connections between CHIP, inflammation, and HFpEF may have implications for future management of HFpEF, including development of targeted therapies.

## Notes

Supplement 1.
eMethods.
**eFigure.** Study Flowchart **eTable 1.** Driver Mutations Queried to Define CHIP in the Present Study **eTable 2.** Multivariable-Adjusted Associations of Clonal Hematopoiesis of Indeterminate Potential (CHIP) and Gene-Specific CHIP Subtypes With Incident HF With Preserved and Reduced Ejection Fraction (HFpEF and HFrEF) Using Models Incorporating Coronary Artery Disease (CAD) and Diabetes as Time-Varying Covariates **eTable 3.** Multivariable-Adjusted Associations of Clonal Hematopoiesis of Indeterminate Potential (CHIP) and Gene-Specific CHIP Subtypes With Incident HF With Preserved and Reduced Ejection Fraction (HFpEF and HFrEF) After Excluding Individuals With Prevalent Coronary Artery Disease (CAD) at Blood Draw **eTable 4.** Multivariable-Adjusted Associations of Clonal Hematopoiesis of Indeterminate Potential (CHIP) and Gene-Specific CHIP Subtypes With Incident HF With Preserved and Reduced Ejection Fraction (HFpEF and HFrEF) in Participants <65 Years **eTable 5.** Multivariable-Adjusted Associations of Clonal Hematopoiesis of Indeterminate Potential (CHIP) and Gene-Specific CHIP Subtypes With Incident HF With Preserved and Reduced Ejection Fraction (HFpEF and HFrEF) in Participants ≥65 Years **eTable 6.** Multivariable-Adjusted Models Testing the Interaction Between Clonal Hematopoiesis of Indeterminate Potential (CHIP) and Age Category (<65 vs. ≥65 Years) on Risk of Incident HF With Preserved and Reduced Ejection Fraction (HFpEF and HFrEF) **eTable 7.** Multivariable-Adjusted Associations of Clonal Hematopoiesis of Indeterminate Potential (CHIP) and Gene-Specific CHIP Subtypes With Variant Allele Fraction (VAF) >10% With Incident HF With Preserved and Reduced Ejection Fraction (HFpEF and HFrEF) **eTable 8.** Multivariable-Adjusted Associations of Clonal Hematopoiesis of Indeterminate Potential (CHIP) With HF With Preserved and Reduced Ejection Fraction (HFpEF and HFrEF) by C-Reactive Protein (CRP) <2 vs. ≥2 mg/L
eReferences

Supplement 2.
Data Sharing Statement
