# Supplementary material for: Clonal Hematopoiesis and Incident Heart Failure With Preserved Ejection Fraction
Source: JAMA Netw Open. 2024 Jan 25;7(1):e2353244. doi: 10.1001/jamanetworkopen.2023.53244 (PMC10811556; doi:10.1001/jamanetworkopen.2023.53244)
Supplement: Supplement 1. — eMethods. eFigure. Study Flowchart eTable 1. Driver Mutations Queried to Define CHIP in the Present Study eTable 2. Multivariable-Adjusted Associations of Clonal Hematopoiesis of Indeterminate Potential (CHIP) and Gene-Specific CHIP Subtypes With Incident HF With Preserved and Reduced Ejection Fraction (HFpEF and HFrEF) Using Models Incorporating Coronary Artery Disease (CAD) and Diabetes as Time-Varying Covariates eTable 3. Multivariable-Adjusted Associations of Clonal Hematopoiesis of Indeterminate Potential (CHIP) and Gene-Specific CHIP Subtypes With Incident HF With Preserved and Reduced Ejection Fraction (HFpEF and HFrEF) After Excluding Individuals With Prevalent Coronary Artery Disease (CAD) at Blood Draw eTable 4. Multivariable-Adjusted Associations of Clonal Hematopoiesis of Indeterminate Potential (CHIP) and Gene-Specific CHIP Subtypes With Incident HF With Preserved and Reduced Ejection Fraction (HFpEF and HFrEF) in Participants <65 Years eTable 5. Multivariable-Adjusted Associations of Clonal Hematopoiesis of Indeterminate Potential (CHIP) and Gene-Specific CHIP Subtypes With Incident HF With Preserved and Reduced Ejection Fraction (HFpEF and HFrEF) in Participants ≥65 Years eTable 6. Multivariable-Adjusted Models Testing the Interaction Between Clonal Hematopoiesis of Indeterminate Potential (CHIP) and Age Category (<65 vs. ≥65 Years) on Risk of Incident HF With Preserved and Reduced Ejection Fraction (HFpEF and HFrEF) eTable 7. Multivariable-Adjusted Associations of Clonal Hematopoiesis of Indeterminate Potential (CHIP) and Gene-Specific CHIP Subtypes With Variant Allele Fraction (VAF) >10% With Incident HF With Preserved and Reduced Ejection Fraction (HFpEF and HFrEF) eTable 8. Multivariable-Adjusted Associations of Clonal Hematopoiesis of Indeterminate Potential (CHIP) With HF With Preserved and Reduced Ejection Fraction (HFpEF and HFrEF) by C-Reactive Protein (CRP) <2 vs. ≥2 mg/L eReferences [file jamanetwopen-e2353244-s001.pdf]

Supplemental Online Content

Schuermans A, Honigberg MC, Raffield LM, et al. Clonal hematopoiesis and incident heart failure with preserved ejection fraction. *JAMA Netw Open*. 2024;7(1):e2353244. doi:10.1001/jamanetworkopen.2023.53244

eMethods.
eFigure. Study Flowchart
eTable 1. Driver Mutations Queried to Define CHIP in the Present Study
eTable 2. Multivariable-Adjusted Associations of Clonal Hematopoiesis of Indeterminate Potential (CHIP) and Gene-Specific CHIP Subtypes With Incident HF With Preserved and Reduced Ejection Fraction (HFpEF and HFrEF) Using Models Incorporating Coronary Artery Disease (CAD) and Diabetes as Time-Varying Covariates
eTable 3. Multivariable-Adjusted Associations of Clonal Hematopoiesis of Indeterminate Potential (CHIP) and Gene-Specific CHIP Subtypes With Incident HF With Preserved and Reduced Ejection Fraction (HFpEF and HFrEF) After Excluding Individuals With Prevalent Coronary Artery Disease (CAD) at Blood Draw
eTable 4. Multivariable-Adjusted Associations of Clonal Hematopoiesis of Indeterminate Potential (CHIP) and Gene-Specific CHIP Subtypes With Incident HF With Preserved and Reduced Ejection Fraction (HFpEF and HFrEF) in Participants <65 Years
eTable 5. Multivariable-Adjusted Associations of Clonal Hematopoiesis of Indeterminate Potential (CHIP) and Gene-Specific CHIP Subtypes With Incident HF With Preserved and Reduced Ejection Fraction (HFpEF and HFrEF) in Participants ≥65 Years
eTable 6. Multivariable-Adjusted Models Testing the Interaction Between Clonal Hematopoiesis of Indeterminate Potential (CHIP) and Age Category (<65 vs. ≥65 Years) on Risk of Incident HF With Preserved and Reduced Ejection Fraction (HFpEF and HFrEF)
eTable 7. Multivariable-Adjusted Associations of Clonal Hematopoiesis of Indeterminate Potential (CHIP) and Gene-Specific CHIP Subtypes With Variant Allele Fraction (VAF) >10% With Incident HF With Preserved and Reduced Ejection Fraction (HFpEF and HFrEF)
eTable 8. Multivariable-Adjusted Associaitons of Clonal Hematopoiesis of Indeterminate Potential (CHIP) With HF With Preserved and Reduced Ejection Fraction (HFpEF and HFrEF) by C-Reactive Protein (CRP) <2 vs. ≥2 mg/L
eReferences

This supplemental material has been provided by the authors to give readers additional information about their work.

© 2024 Schuermans A et al. *JAMA Network Open.*

eMethods

Jackson Heart Study (JHS)

The JHS is a prospective cohort including 5,306 self-identified Black adults aged 21 to 84 years from Jackson, Mississippi, and the surrounding area. The JHS was designed as a community-based cohort to investigate risk factors for cardiovascular diseases in self-identified Black U.S. citizens. Written informed consent was obtained from all study participants. The study protocol was approved by institutional review boards from the University of Mississippi Medical Center, Jackson State University, and Tougaloo College.

At enrolment (2000-2004) and subsequent study visits (2005-2009 and 2009-2013), participants underwent physical examination (including anthropometry and blood pressure measurements), donated blood samples, and provided detailed information on lifestyle, environment, and health history. We considered JHS participants for inclusion if they had whole-genome sequences available *(N*=3,404), which were generated as part of the NHLBI TOPMed program (National Heart, Lung, and Blood Institute Trans-Omics for Precision Medicine) in 3,404 consenting participants. Blood samples for whole-genome sequencing were collected at enrolment (*n*=3,284; 96.5%) or the second study visit (*n*=120; 3.5%). Covariate data were obtained from each participant's corresponding study visit, except for smoking status, income, and education, which were only evaluated at baseline. Smoking was self-reported and categorized as (1) *Current*; (2) *Previous*; (3) *Never*; or (4) *Missing*. Income categories were assigned based on family income and size and were adjusted for year of data collection to account for inflation, with (1) *Less than the poverty level*; (2) *Lower-middle (1 to 1.5 times the poverty level)*; (3) *Upper-middle (1.5 to 3.5 times the poverty level)*; (4) *Affluent (>3.5 times the poverty level)*; and (5) *Missing* as the different categories. Education was incorporated as a four-category variable, with (1) *Less than high school*; (2) *High school (or General Educational Development test) graduate*; (3); *Attended vocational school, trade school, or college*; and (4) *Missing* as the different categories.

Potential heart failure (HF) hospitalizations in the JHS were identified and adjudicated as described previously.[1] In brief, hospitalization data were obtained from hospital discharge lists and annual follow-up interviews. Self-reported data from the follow-up interviews were verified using data from hospital discharge lists. Hospital diagnoses based on ICD codes (*International Classification of Diseases*) were reviewed by trained personnel and adjudicated using the available clinical data (including clinical notes, laboratory tests, and imaging data). HF events were classified as HF with preserved ejection fraction (HFpEF; left ventricular ejection fraction ≥50%) or HF with reduced ejection fraction (HFrEF; left ventricular ejection fraction <50%) based on imaging data before and at the time of the initial HF hospitalization. JHS participants were followed for incident HF events from 2005 through 2016.

© 2024 Schuermans A et al. *JAMA Network Open.*

.

Women's Health Initiative (WHI)

The WHI is a prospective, multicenter study including 161,808 postmenopausal women recruited at 40 centers throughout the U.S. between 1993 and 1998. WHI participants enrolled in an observational study or at least one of three clinical trials (hormone therapy, calcium/vitamin D supplementation, or dietary modification). All WHI participants provided informed consent, and study protocols were approved by the appropriate local institutional review boards.

At enrolment, all included participants underwent physical examination (including anthropometry and blood pressure measurements) and provided information on medical history, health-related behaviors, and socio-economic characteristics. Race/ethnicity was ascertained using self-report, categorized as (1) *American Indian or Alaskan Native*; (2) *Asian or Pacific Islander*; (3) *Black or African-American*; (4) *Hispanic/Latino*; (5) *White (not of Hispanic origin)*; and (6) *Other*. Smoking was self-reported and categorized as (1) *Current*; (2) *Previous*; (3) *Never*; or (4) *Missing*. Income was incorporated as a categorical variable based on household income, with (1) *<$20,000*; (2) *$20,000-34,999*; (3) *$35,000-49,999*; (4) $*50,000-74,999*; (5) *>$75,000*; and (6) *Missing* as the different categories. Education was incorporated as a five-category variable, with (1) *Not graduated from high school*; (2) *High school*; (3) *Some college or trade school*; (4) *College or more advanced*; and (5) *Missing* as the different categories.

Of the 161,808 women in the original cohort, a sub-cohort of 44,174 women, were followed prospectively from baseline for hospitalized HF events. The WHI HF subcohort comprises a subset of participants randomized to the WHI hormone trial (*n*=27,347), as well as all Black and Hispanic participants from the observational study and other clinical trial components (*n*=16,827). Participants included in the WHI HF subcohort were followed for HF adjudication by yearly medical record abstraction of hospitalizations.[2] Trained adjudicators evaluated all confirmed HF hospitalizations, as well as patient-reported development of HF, angina, or other cardiovascular diseases, by reviewing clinical data (including clinical notes, laboratory tests, and imaging data) for evidence of HF. HF events were classified as HFpEF (left ventricular ejection fraction ≥50%) or HFrEF (left ventricular ejection fraction <50%) based on imaging data before and at the time of the initial HF hospitalization. WHI participants were followed for incident HF events from enrolment through 2022.

A subsample of unrelated WHI participants aged 50-79 years at blood draw had available CHIP calls from whole-genome sequences through the NHLBI TOPMed program (*n*=11,010; January 2020). Participants included in the WHI TOPMed sample were selected from a nested case-control study of incident stroke and venous thromboembolism. A subset

of 3,643 WHI participants included in the current CHIP-HF analysis had available C-reactive protein measurements at the baseline exam, including 2,705 measured at the University of Minnesota (Modular P, Roche Diagnostics) and 938 at the University of Vermont (BN II, Dade Behring).

All multivariable-adjusted WHI models included inverse probability weights to account for inclusion in the TOPMed cohort as well as inclusion in the WHI HF cohort to allow inference to the sampling frame from which the participants was drawn, a process that decreases parameter bias.[3]

© 2024 Schuermans A et al. *JAMA Network Open.*

eFigure. Study flowchart.

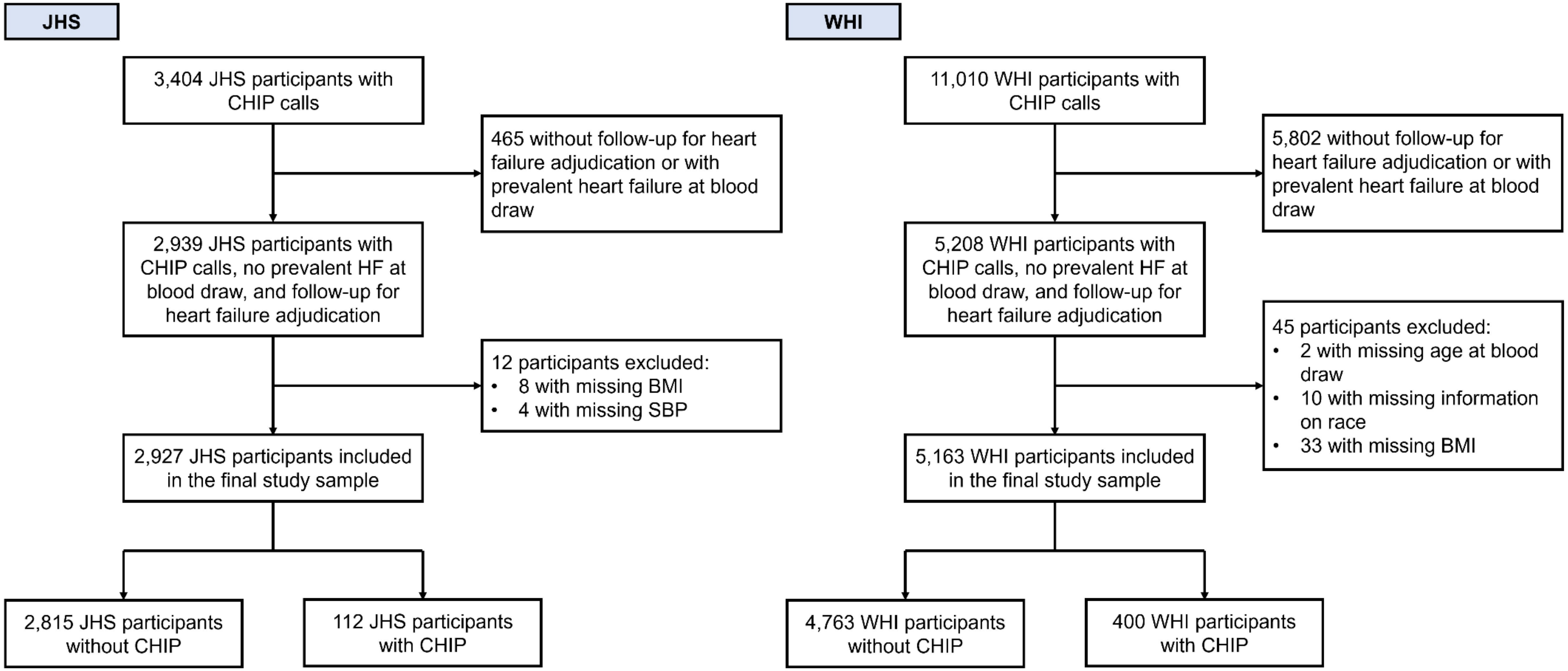

BMI indicates body mass index; CHIP, clonal hematopoiesis of indeterminate potential; HF, heart failure; JHS, Jackson Heart Study; SBP, systolic blood pressure; WHI, Women's Health Initiative.

© 2024 Schuermans A et al. *JAMA Network Open.*

eTable 1. Driver mutations queried to define CHIP in the present study.

Gene name	Reported mutations used for variant calling
ASXL1	Frameshift/nonsense/splice-site in exon 11-12
ASXL2	Frameshift/nonsense/splice-site in exon 11-12
BCOR	Frameshift/nonsense/splice-site
BCORL1	Frameshift/nonsense/splice-site
BRAF	G464E, G464V, G466E, G466V, G469R, G469E, G469A, G469V, V471F, V472S, L485W, N581S, I582M, I592M, I592V, D594N, D594G, D594V, D594E, F595L, F595S, G596R, L597V, L597S, L597Q, L597R, A598V, V600M, V600L, V600K, V600R, V600E, V600A, V600G, V600D, K601E, K601N, R603*, W604R, W604G, S605G, S605F, S605N, G606E, G606A, G606V, H608R, H608L, G615R, S616P, S616F, L618S, L618W
BRCC3	Frameshift/nonsense/splice-site
CBL	RING finger missense p.381-421
CBLB	RING finger missense p.372-412
CEBPA	Frameshift/nonsense/splice-site
CREBBP	Frameshift/nonsense/splice-site, D1435E, R1446L, R1446H, R1446C, Y1450C, P1476R, Y1482H, H1487Y, W1502C, Y1503D, Y1503H, Y1503F, S1680del
CSF1R	L301F, L301S, Y969C, Y969N, Y969F, Y969H, Y969D
CSF3R	T615A, T618I, truncating c.741-791
CTCF	Frameshift/nonsense, R377C, R377H, P378A, P378L
CUX1	Frameshift/nonsense
DNMT3A	Frameshift/nonsense/splice-site, F290I, F290C, V296M, P307S, P307R, R326H, R326L, R326C, R326S, G332R, G332E, V339A, V339M, V339G, L344Q, L344P, R366P, R366H, R366G, A368T, A368V, R379H, R379C, I407T, I407N, I407S, F414L, F414S, F414C, A462V, K468R, C497G, C497Y, Q527H, Q527P, Y533C, S535F, C537G, C537R, G543A, G543S, G543C, L547H, L547P, L547F, M548I, M548K, G550R, W581R, W581G, W581C, R604Q, R604W, R635W, R635Q, S638F, G646V, G646E, L653W, L653F, I655N, V657A, V657M, R659H, Y660C, V665G, V665L, M674V, R676W, R676Q, G685R, G685E, G685A, D686Y, D686G, R688H, G699R, G699S, G699D, P700L, P700S, P700R, P700Q, P700T, P700A, D702N, D702Y, V704M, V704G, I705F, I705T, I705S, I705N, G707D, G707V, C710S, C710Y, S714C, V716D, V716F, V716I, N717S, N717I, P718L, R720H, R720G, K721R, K721T, Y724C, R729Q, R729W, R729G, F731C, F731L, F731Y, F731I, F732del, F732C, F732S, F732L, E733G, E733A, F734L, F734C, Y735C, Y735N, Y735S, R736H, R736C, R736P, L737H, L737V, L737F, L737R, A741V, P742P, P743R, P743L, R749C, R749L, R749H, R749G, F751L, F751C, F752del, F752C, F752L, F752I, F752V, W753G, W753C, W753R, L754P, L754R, L754H,

© 2024 Schuermans A et al. *JAMA Network Open.*

	F755S, F755I, F755L, M761I, M761V, G762C, V763I, S770L, S770W, S770P, R771Q, F772I, F772V, L773R, L773V, E774K, E774D, E774G, I780T, D781G, R792H, W795C, W795L, G796D, G796V, N797Y, N797H, N797S, P799S, P799R, P799H, R803S, R803W, P804L, P804S, K826R, S828N, K829R, T835M, N838D, K841Q, Q842E, P849L, D857N, W860R, E863D, F868S, G869S, G869V, M880V, S881R, S881I, R882H, R882P, R882C, R882G, A884P, A884V, Q886R, L889P, L889R, G890D, G890R, G890S, V895M, P896L, V897G, V897D, R899L, R899H, R899C, L901R, L901H, P904L, F909C, P904Q, A910P, C911R, C911Y
EED	Frameshift/nonsense/splice-site, L240Q, I363M
EP300	Frameshift/nonsense/splice_site, VF1148_1149del, D1399N, D1399Y, P1452L, Y1467N, Y1467H, Y1467C, R1627W, A1629V
ETNK1	N244S, N244T, N244K
ETV6	Frameshift/nonsense/splice-site
EZH2	Frameshift/nonsense/splice-site, Q62R, N102S, F145S, F145C, F145Y, F145L, G159R, E164D, R202Q, K238E, E244K, R283Q, H292R, P488S, R497Q, R561H, T568I, K629E, Y641N, Y641H, Y641S, Y641C, Y641F, D659Y, D659G, V674M, A677G, A677V, R679C, R679H, R685C, R685H, A687V, N688I, N688K, H689Y, S690P, I708V, I708T, I708M, E720K, E740K
FLT3	V579A, V592A, V592I, F594L, FY590-591GD, D835Y, D835H, D835E, del835
GATA1	Frameshift/nonsense/splice-site
GATA2	Frameshift/nonsense/splice-site, R293Q, N317H, A318T, A318V, A318G, G320D, L321P, L321F, L321V, Q328P, R330Q, R361L, L359V, A372T, R384G, R384K
GATA3	Frameshift/nonsense/splice-site ZNF domain, R276W, R276Q, N286T, L348V,
GNA13	I34T, G57S, S62F, M68K, Q134R, Y145F, L152F, E167D, Q169H, R264H, E273K, V322G, V362G, L371F
GNAS	R201S, R201C, R201H, R201L, Q227K, Q227R, Q227L, Q227H, R374C
GNB1	K57N, K57M, K57E, K57T, I80T, I80N
IDH1	R132C, R132G, R132H, R132L, R132P, R132V, V178I
IDH2	R140W, R140Q, R140L, R140G, R172W, R172G, R172K, R172T, R172M, R172N, R172S
IKZF1	Frameshift/nonsense
IKZF2	Frameshift/nonsense
IKZF3	Frameshift/nonsense
JAK1	T478A, T478S, V623A, A634D, L653F, R724H, R724Q, R724P, T782M, L783F
JAK2	N533D, N533Y, N533S, H538R, K539E, K539L, I540T, I540V, V617F, R683S, R683G, del/ins537-539L, del/ins538-539L, del/ins540-543MK, del/ins540-544MK, del/ins541-543K, del542-543, del543-544, ins11546-547
JAK3	M511T, M511I, A572V, A572T, A573V, R657Q, V715I, V715A
KDM6A	Frameshift/nonsense/splice-site, del419

© 2024 Schuermans A et al. *JAMA Network Open.*

KIT	ins503, V559A, V559D, V559G, V559I, V560D, V560A, V560G, V560E, del560, E561K, del579, P627L, P627T, R634W, K642E, K642Q, V654A, V654E, H697Y, H697D, E761D, K807R, D816H, D816Y, D816F, D816I, D816V, D816H, del551-559
KRAS	G12D, G12A, G12E, G12V, G13D, G13C, G13Y, G13F, G13R, G13A, G13V, G13E, V14I, T58I, G60D, G60A, G60V, Q61K, Q61E, Q61P, Q61R, Q61L, Q61H, K117E, K117N, A146T, A146P, A146V
LUC7L2	Frameshift/nonsense/splice-site
MLL	Frameshift/nonsense
MLL2	Frameshift/nonsense
MPL	S505G, S505N, S505C, L510P, del513, W515A, W515R, W515K, W515S, W515L, A519T, A519V, Y591D, W515-518KT
NF1	Frameshift/nonsense
NPM1	Frameshift p.W288fs (insertion at c.859_860, 860_861, 862_863, 863_864)
NRAS	G12S, G12R, G12C, G12N, G12P, G12Y, G12D, G12A, G12V, G12E, G13S, G13R, G13C, G13N, G13P, G13Y, G13D, G13A, G13V, G13E, G60E, G60R, Q61R, Q61L, Q61K, Q61P, Q61H, Q61Q
PDS5B	Frameshift/nonsense/splice-site, R1292Q
PDSS2	Frameshift/nonsense
PHF6	Frameshift/nonsense/splice-site, A40D, M125I, S246Y, F263L, R274Q, C297Y, H302Y, H329L
PHIP	Frameshift/nonsense/splice-site
PPM1D	Frameshift/nonsense, exon 5 or 6
PRPF40B	Frameshift/nonsense/splice-site, P15H, M58I, P405L, P562S,
PRPF8	M1307I, C1594W, D1598Y, D1598N, D1598V
PTEN	Frameshift/nonsense/splice-site, D24G, R47G, F56V, L57W, H61R, K66N, Y68H, C71Y, F81C, Y88C, D92G, D92V, D92E, H93Y, H93D, H93Q, N94I, P95L, I101T, C105F, C105S, D107Y, L112V, H123Y, C124R, C124S, K125E, A126D, K128N, R130G, R130Q, R130L, G132D, I135V, I135K, C136R, C136F, K144Q, A151T, D153Y, D153N, Y155H, Y155C, R159K, R159S, R161K, R161I, G165R, G165E, S170N, S170I, R173C, Y174D, Y177C, H196Y, R234W, G251C, D252Y, F271S, D326G
PTPN11	G60V, G60R, G60A, D61Y, D61V, D61G, Y63C, E69K, E69G, E69D, E69Q, F71L, F71K, A72T, A72V, A72D, T73I, E76K, E76Q, E76M, E76A, E76G, E139G, E139D, N308D, N308T, N339S, P491L, S502P, S502A, S502L, G503V, G503G, G503A, G503E, Q506P, T507A, T507K
RAD21	Frameshift/nonsense/splice-site, R65Q, H208R, Q474R
RUNX1	Frameshift/nonsense/splice-site, S73F, H78Q, H78L, R80C, R80P, R80H, L85Q, P86L, P86H, S114L, D133Y, L134P, R135G, R135K, R135S, R139Q, R142S, A165V, R174Q, R177L, R177Q, A224T, D171G, D171V, D171N, R205W, R223C
SETBP1	D868N, D868T, S869N, G870S, I871T, D880N, D880Q

© 2024 Schuermans A et al. *JAMA Network Open.*

SETD2	Frameshift/nonsense, V1190M
SETDB1	Frameshift/nonsense, K715E
SF1	Frameshift/nonsense/splice-site, T454M, Y476C, A508G
SF3A1	Frameshift/nonsense/splice-site, A57S, M117I, K166T, Y271C
SF3B1	G347V, R387W, R387Q, E592K, E622D, Y623C, R625L, R625C, R625G, H662Q, H662D, T663I, K666N, K666T, K666E, K666R, K700E, V701F, A708T, G740R, G740E, A744P, D781G, E783K, R831Q, L833F, E862K, R957Q
SRSF2	Y44H, P95H, P95L, P95T, P95R, P95A, P107H, P95fs
SMC1A	K190T, R586W, M689V, R807H, R1090H, R1090C
SMC3	Frameshift/nonsense, R155I, Q367E, D392V, K571R, R661P, G662C
STAG1	Frameshift/nonsense/splice-site, H1085Y
STAG2	Frameshift/nonsense/splice-site
SUZ12	Frameshift/nonsense
TET2	Frameshift/nonsense/splice-site, missense mutations in catalytic domains (p.1104-1481 and 1843-2002)
TP53	Frameshift/nonsense/splice-site, S46F, G105C, G105R, G105D, G108S, G108C, R110L, R110C, T118A, T118R, T118I, S127F, S127Y, L130V, L130F, K132Q, K132E, K132W, K132R, K132M, K132N, F134V. F134L, F134S, C135W, C135S, C135F, C135G, C135Y, Q136K, Q136E, Q136P, Q136R, Q136L, Q136H, A138P, A138V, A138A, A138T, T140I, C141R, C141G, C141A, C141Y, C141S, C141F, C141W, V143M, V143A, V143E, L145Q, W146C, W146L, L145R, V147G, P151T, P151A, P151S, P151H, P151R, P152S, P152R, P152L, T155P, T155A, V157F, R158H, R158L, A159V, A159P, A159S, A159D, A161T, A161D, Y163N, Y163H, Y163D, Y163S, Y163C, K164E, K164M, K164N, K164P, H168Y, H168P, H168R, H168L, H168Q, M169I, M169T, M169V, E171K, E171Q, E171G, E171A, E171V, E171D, V172D, V173M, V173L, V173G, R174W, R175G, R175C, R175H, C176R, C176G, C176Y, C176F, C176S, P177R, P177R, P177L, H178D, H178P, H178Q, H179Y, H179R, H179Q, R181C, R181Y, D186G, G187S, P190L, P190T, H193N, H193P, H193L, H193R, L194F, L194R, I195F, I195N, I195T, R196P, V197L, G199V, Y205N, Y205C, Y205H, D208V, R213Q, R213P, R213L, R213Q, H214D, H214R, S215G, S215I, S215R, V216M, V217G, Y220N, Y220H, Y220S, Y220C, E224D, I232F, I232N, I232T, I232S, Y234N, Y234H, Y234S, Y234C, Y236N, Y236H, Y236C, M237V, M237K, M237I, C238R, C238G, C238Y, C238W, N239T, N239S, S241Y, S241C, S241F, C242G, C242Y, C242S, C242F, G244S, G244C, G244D, G245S, G245R, G245C, G245D, G245A, G245V, G245S, M246V, M246K, M246R, M246I, N247I, R248W, R248G, R248Q, R249G, R249W, R249T, R249M, P250L, I251N, L252P, I254S, I255F, I255N, I255S, L257Q, L257P, E258K, E258Q, D259Y, S261T, G262D, G262V, L265P, G266R, G266E, G266V, R267W, R267Q, R267P, E271K, V272M, V272L, R273S, R273G, R273C, R273H, R273P, R273L, V274F, V274D, V274A, V274G, V274L, C275Y, C275S, C275F, A276P, C277F, C277Y, P278T, P278A, P278S, P278H, P278R, P278L, G279E, R280G, R280K, R280T, R280I, R280S, D281N, D281H, D281Y, D281G, D281E, R282G, R282W, R282Q, R282P, E285K, E285V, E286G, E286V, E286K, K320N, L330R, G334V, R337C, R337L, A347T, L348F, T377P

© 2024 Schuermans A et al. *JAMA Network Open.*

U2AF1	D14G, S34F, S34Y, R35L, R156H, R156Q, Q157R, Q157P
U2AF2	R18W, Q143L, M144I, L187V, Q190L
WT1	Frameshift/nonsense/splice-site
ZRSR2	Frameshift/nonsense, R126P, E133G, C181F, H191Y, I202N, F239V, F239Y, N261Y, C280R, C302R, C326R, H330R, N382K

© 2024 Schuermans A et al. *JAMA Network Open.*

eTable 2. Multivariable-adjusted associations of clonal hematopoiesis of indeterminate potential (CHIP) and gene-specific CHIP subtypes with incident HF with preserved and reduced ejection fraction (HFpEF and HFrEF) using models incorporating coronary artery disease (CAD) and diabetes as time-varying covariates.

	Jackson Heart Study			Women's Health Initiative			Meta-analysis			
	**Cases / total *n***	**HR (95% CI)**	***P*-value**	**Cases / total *n***	**HR (95% CI)**	***P*-value**	**HR (95% CI)**	***P*-value**	***P*-value for between-study heterogeneity (Cochran *Q*)**	**I^2 statistic (%)**
**Any HF**	-	-	-	-	-	-	-	-	-	-
Any CHIP	18 / 112	1.25 (0.80 to 1.98)	0.33	65 / 400	1.15 (0.90 to 1.46)	0.264	1.17 (0.95 to 1.45)	0.15	0.73	0.0
DNMT3A CHIP	12 / 68	1.37 (0.79 to 2.38)	0.26	34 / 245	0.94 (0.67 to 1.30)	0.689	1.03 (0.78 to 1.37)	0.82	0.24	26.4
TET2 CHIP	6 / 21	3.24 (1.51 to 6.97)	0.003	20 / 87	1.85 (1.23 to 2.79)	0.003	2.1 (1.46 to 3.01)	<0.001	0.21	37.6
**HFpEF**	-	-	-	-	-	-	-	-	-	-
Any CHIP	8 / 112	1.19 (0.62 to 2.30)	0.60	35 / 400	1.25 (0.90 to 1.73)	0.192	1.23 (0.92 to 1.66)	0.16	0.91	0.0
DNMT3A CHIP	4 / 68	0.96 (0.38 to 2.37)	0.92	20 / 245	1.13 (0.74 to 1.73)	0.57	1.1 (0.75 to 1.61)	0.64	0.74	0.0
TET2 CHIP	3 / 21	3.83 (1.37 to 10.69)	0.01	10 / 87	1.75 (0.95 to 3.20)	0.07	2.14 (1.27 to 3.61)	0.004	0.20	40.0
**HFrEF**	-	-	-	-	-	-	-	-	-	-
Any CHIP	5 / 112	0.76 (0.30 to 1.88)	0.55	14 / 400	0.72 (0.43 to 1.19)	0.198	0.73 (0.47 to 1.13)	0.16	0.92	0.0
DNMT3A CHIP	3 / 68	0.74 (0.23 to 2.38)	0.61	4 / 245	0.29 (0.11 to 0.77)	0.013	0.43 (0.2 to 0.91)	0.03	0.23	32.0
TET2 CHIP	2 / 21	2.14 (0.52 to 8.82)	0.29	5 / 87	1.55 (0.73 to 3.31)	0.252	1.67 (0.86 to 3.25)	0.13	0.79	0.0

Hazard ratios (HRs) with corresponding 95% confidence intervals (CIs) were calculated using Cox proportional hazards models adjusted for age at blood draw, income, education, smoking, body mass index, systolic blood draw, antihypertensive medication use, CAD, diabetes mellitus,

© 2024 Schuermans A et al. *JAMA Network Open.*

and stroke. CAD and diabetes mellitus were incorporated as time-varying covariates using the *tmerge()* function in R. JHS models were further adjusted for sex, while WHI models were further adjusted for race/ethnicity and inverse probability weights for inclusion in the TOPMed and HF adjudication cohorts. Any HF was defined as a composite outcome including HFpEF, HFrEF, and HF with unknown ejection fraction (HFuEF). Effect estimates from both cohorts were meta-analyzed using fixed effects. Follow-up in the WHI was truncated at 22 years due to deviations from proportional hazards. Follow-up occurred over a median of 12.0 (IQR: 11.0-12.0) years in the JHS and 15.3 (IQR: 9.0-22.0) years in the WHI.

© 2024 Schuermans A et al. *JAMA Network Open.*

eTable 3. Multivariable-adjusted associations of clonal hematopoiesis of indeterminate potential (CHIP) and gene-specific CHIP subtypes with incident HF with preserved and reduced ejection fraction (HFpEF and HFrEF) after excluding individuals with prevalent coronary artery disease (CAD) at blood draw.

	Jackson Heart Study			Women's Health Initiative			Meta-analysis			
	**Cases / total *n***	**HR (95% CI)**	***P*-value**	**Cases / total *n***	**HR (95% CI)**	***P*-value**	**HR (95% CI)**	***P*-value**	***P*-value for between-study heterogeneity (Cochran *Q*)**	***I*² statistic (%)**
**Any HF**	-	-	-	-	-	-	-	-	-	-
Any CHIP	17 / 106	1.33 (0.81 to 2.21)	0.26	60 / 385	1.24 (0.95 to 1.62)	0.11	1.26 (0.99 to 1.6)	0.06	0.80	0.0
DNMT3A CHIP	12 / 66	1.47 (0.81 to 2.66)	0.21	31 / 234	1.03 (0.72 to 1.49)	0.86	1.14 (0.83 to 1.55)	0.42	0.33	0.0
TET2 CHIP	5 / 18	4.02 (1.61 to 10.02)	0.003	20 / 87	1.97 (1.25 to 3.08)	0.003	2.26 (1.51 to 3.39)	<0.001	0.17	47.2
**HFpEF**	-	-	-	-	-	-	-	-	-	-
Any CHIP	7 / 106	1.06 (0.49 to 2.31)	0.87	34 / 385	1.4 (0.97 to 2.00)	0.07	1.33 (0.96 to 1.84)	0.09	0.54	0.0
DNMT3A CHIP	4 / 66	0.95 (0.34 to 2.62)	0.92	19 / 234	1.23 (0.77 to 1.97)	0.38	1.18 (0.77 to 1.8)	0.45	0.65	0.0
TET2 CHIP	2 / 18	3.86 (0.91 to 16.29)	0.07	10 / 87	2.06 (1.09 to 3.89)	0.03	2.28 (1.27 to 4.09)	0.006	0.43	0.0
**HFrEF**	-	-	-	-	-	-	-	-	-	-
Any CHIP	5 / 106	1.07 (0.43 to 2.67)	0.89	12 / 385	0.75 (0.42 to 1.35)	0.34	0.83 (0.51 to 1.37)	0.47	0.53	0.0
DNMT3A CHIP	3 / 66	0.96 (0.3 to 3.09)	0.94	4 / 234	0.42 (0.16 to 1.13)	0.09	0.59 (0.28 to 1.26)	0.17	0.29	10.2
TET2 CHIP	2 / 18	3.07 (0.73 to 12.98)	0.13	5 / 87	1.43 (0.58 to 3.49)	0.43	1.77 (0.83 to 3.77)	0.14	0.38	0.0

Hazard ratios (HRs) with corresponding 95% confidence intervals (CIs) were calculated using Cox proportional hazards models adjusted for age at blood draw, income, education, smoking, body mass index, systolic blood draw, antihypertensive medication use, prevalent diabetes mellitus,

© 2024 Schuermans A et al. *JAMA Network Open.*

and stroke. JHS models were further adjusted for sex, while WHI models were further adjusted for race/ethnicity and inverse probability weights for inclusion in the TOPMed and HF adjudication cohorts. Any HF was defined as a composite outcome including HFpEF, HFrEF, and HF with unknown ejection fraction (HFuEF). Effect estimates from both cohorts were meta-analyzed using fixed effects. Follow-up in the WHI was truncated at 22 years due to deviations from proportional hazards. There were a total of 2,826 JHS and 4,941 WHI participants without prevalent CAD at blood draw. Follow-up occurred over a median (IQR) of 12.0 (11.2-12.0) years in the JHS and 15.5 (9.1-22.0) years in the WHI.

© 2024 Schuermans A et al. *JAMA Network Open.*

eTable 4. Multivariable-adjusted associations of clonal hematopoiesis of indeterminate potential (CHIP) and gene-specific CHIP subtypes with incident HF with preserved and reduced ejection fraction (HFpEF and HFrEF) in participants <65 years.

	Jackson Heart Study			Women's Health Initiative			Meta-analysis			
	**Cases / total *n***	**HR (95% CI)**	***P*-value**	**Cases / total *n***	**HR (95% CI)**	***P*-value**	**HR (95% CI)**	***P*-value**	***P*-value for between-study heterogeneity (Cochran *Q*)**	***I*² statistic (%)**
**Any HF**	-	-	-	-	-	-	-	-	-	-
Any CHIP	4 / 52	1.22 (0.44 to 3.39)	0.70	8 / 100	1.10 (0.53 to 2.26)	0.80	1.14 (0.63 to 2.05)	0.67	0.87	0.0
DNMT3A CHIP	3 / 36	1.49 (0.46 to 4.79)	0.50	3 / 61	0.63 (0.2 to 2)	0.44	0.96 (0.42 to 2.19)	0.93	0.31	4.4
TET2 CHIP	1 / 8	1.07 (0.14 to 7.94)	0.95	3 / 21	2.10 (0.65 to 6.78)	0.21	1.77 (0.64 to 4.87)	0.27	0.57	0.0
**HFpEF**	-	-	-	-	-	-	-	-	-	-
Any CHIP	3 / 52	1.73 (0.51 to 5.85)	0.38	2 / 100	0.69 (0.17 to 2.87)	0.61	1.17 (0.46 to 2.96)	0.74	0.34	0.0
DNMT3A CHIP	2 / 36	1.81 (0.42 to 7.71)	0.43	0 / 61	-	-	1.81 (0.42 to 7.71)	0.43	>0.99	0.0
TET2 CHIP	1 / 8	2.09 (0.25 to 17.68)	0.50	1 / 21	1.72 (0.23 to 12.97)	0.60	1.88 (0.43 to 8.18)	0.40	0.90	0.0
**HFrEF**	-	-	-	-	-	-	-	-	-	-
Any CHIP	1 / 52	0.70 (0.09 to 5.22)	0.73	4 / 100	1.06 (0.38 to 2.99)	0.91	0.97 (0.39 to 2.44)	0.95	0.72	0.0
DNMT3A CHIP	1 / 36	1.22 (0.16 to 9.11)	0.85	1 / 61	0.42 (0.06 to 3.08)	0.39	0.71 (0.17 to 2.93)	0.64	0.46	0.0
TET2 CHIP	0 / 8	-	-	2 / 21	2.9 (0.67 to 12.6)	0.15	2.90 (0.67 to 12.6)	0.15	>0.99	0.0

Hazard ratios (HRs) with corresponding 95% confidence intervals (CIs) were calculated using Cox proportional hazards models adjusted for age at blood draw, income, education, smoking, body mass index, systolic blood draw, antihypertensive medication use, prevalent CAD, prevalent

© 2024 Schuermans A et al. *JAMA Network Open.*

diabetes mellitus, and stroke. JHS models were further adjusted for sex, while WHI models were further adjusted for race/ethnicity and inverse probability weights for inclusion in the TOPMed and HF adjudication cohorts. Any HF was defined as a composite outcome including HFpEF, HFrEF, and HF with unknown ejection fraction (HFuEF). Effect estimates from both cohorts were meta-analyzed using fixed effects. Follow-up in the WHI was truncated at 22 years due to deviations from proportional hazards. There were a total of 717 JHS and 1,834 WHI participants aged <65 years. Follow-up occurred over a median (IQR) of 12.0 (11.4-12.0) years in the JHS and 20.0 (11.8-22.0) years in the WHI.

© 2024 Schuermans A et al. *JAMA Network Open.*

eTable 5. Multivariable-adjusted associations of clonal hematopoiesis of indeterminate potential (CHIP) and gene-specific CHIP subtypes with incident HF with preserved and reduced ejection fraction (HFpEF and HFrEF) in participants ≥65 years.

	Jackson Heart Study			Women's Health Initiative			Meta-analysis			
	Cases / total *n*	HR (95% CI)	*P*-value	Cases / total *n*	HR (95% CI)	*P*-value	HR (95% CI)	*P*-value	*P*-value for between-study heterogeneity (Cochran *Q*)	I^2 statistic (%)
**Any HF**	-	-	-	-	-	-	-	-	-	-
Any CHIP	14 / 60	1.24 (0.70 to 2.17)	0.46	57 / 300	1.28 (0.97 to 1.68)	0.09	1.27 (0.99 to 1.63)	0.06	0.92	0.0
DNMT3A CHIP	9 / 32	1.30 (0.64 to 2.62)	0.46	31 / 184	1.1 (0.76 to 1.58)	0.62	1.14 (0.82 to 1.58)	0.43	0.68	0.0
TET2 CHIP	5 / 13	5.53 (2.13 to 14.34)	<0.001	17 / 66	1.82 (1.12 to 2.97)	0.02	2.3 (1.49 to 3.55)	<0.001	0.04	75.8
**HFpEF**	-	-	-	-	-	-	-	-	-	-
Any CHIP	5 / 60	0.91 (0.36 to 2.29)	0.83	33 / 300	1.48 (1.02 to 2.14)	0.04	1.38 (0.98 to 1.95)	0.06	0.34	0.0
DNMT3A CHIP	2 / 32	0.58 (0.14 to 2.43)	0.45	20 / 184	1.4 (0.88 to 2.23)	0.15	1.29 (0.83 to 2.01)	0.25	0.25	24.9
TET2 CHIP	2 / 13	5.38 (1.18 to 24.54)	0.03	9 / 66	1.98 (1.01 to 3.88)	0.05	2.33 (1.26 to 4.32)	0.007	0.24	28.1
**HFrEF**	-	-	-	-	-	-	-	-	-	-
Any CHIP	4 / 60	0.89 (0.31 to 2.50)	0.82	10 / 300	0.69 (0.37 to 1.32)	0.27	0.74 (0.43 to 1.28)	0.29	0.69	0.0
DNMT3A CHIP	2 / 32	0.71 (0.17 to 3.03)	0.65	3 / 184	0.34 (0.11 to 1.06)	0.06	0.45 (0.18 to 1.1)	0.08	0.43	0.0
TET2 CHIP	2 / 13	4.75 (1.08 to 20.99)	0.04	3 / 66	0.96 (0.31 to 3.03)	0.95	1.75 (0.7 to 4.33)	0.23	0.10	64.0

Hazard ratios (HRs) with corresponding 95% confidence intervals (CIs) were calculated using Cox proportional hazards models adjusted for age at blood draw, income, education, smoking, body mass index, systolic blood draw, antihypertensive medication use, prevalent CAD, prevalent diabetes mellitus, stroke, CHIP/gene-specific CHIP subtype, and age category (<65 vs. ≥65 years). JHS models were further adjusted for sex,

© 2024 Schuermans A et al. *JAMA Network Open.*

while WHI models were further adjusted for race/ethnicity and inverse probability weights for inclusion in the TOPMed and HF adjudication cohorts. Any HF was defined as a composite outcome including HFpEF, HFrEF, and HF with unknown ejection fraction (HFuEF). Effect estimates from both cohorts were meta-analyzed using fixed effects. Follow-up in the WHI was truncated at 22 years due to deviations from proportional hazards. There were a total of 2,210 JHS and 3,329 WHI participants aged ≥65 years. Follow-up occurred over a median (IQR) of 11.6 (6.1-12.0) years in the JHS and 13.5 (8.0-19.6) years in the WHI.

© 2024 Schuermans A et al. *JAMA Network Open.*

eTable 6. Multivariable-adjusted models testing the interaction between clonal hematopoiesis of indeterminate potential (CHIP) and age category (<65 vs. ≥65 years) on risk of incident HF with preserved and reduced ejection fraction (HFpEF and HFrEF).

	**Jackson Heart Study**	**Women's Health Initiative**	**Meta-analysis**		
	***P*-value (for interaction)**	***P*-value (for interaction)**	***P*-value (for interaction)**	***P*-value for between-study heterogeneity (Cochran *Q*)**	**I^2 statistic (%)**
**Any HF**	-	-	-	-	-
Any CHIP	0.83	0.60	0.58	0.91	0.0
DNMT3A CHIP	0.99	0.39	0.53	0.56	0.0
TET2 CHIP	0.14	0.82	0.59	0.16	49.1
**HFpEF**	-	-	-	-	-
Any CHIP	0.50	0.23	0.70	0.19	42.6
DNMT3A CHIP	0.34	0.99	0.34	0.99	0.0
TET2 CHIP	0.58	0.85	0.61	0.76	0.0
**HFrEF**	-	-	-	-	-
Any CHIP	0.88	0.41	0.51	0.60	0.0
DNMT3A CHIP	0.7	0.76	0.63	0.95	0.0
TET2 CHIP	>0.99	0.18	0.18	>0.99	0.0

P-values were calculated using Cox proportional hazards models adjusted for age at blood draw, income, education, smoking, body mass index, systolic blood draw, antihypertensive medication use, prevalent CAD, prevalent diabetes mellitus, and stroke. JHS models were further adjusted for sex, while WHI models were further adjusted for race/ethnicity and inverse probability weights for inclusion in the TOPMed and HF

© 2024 Schuermans A et al. *JAMA Network Open.*

adjudication cohorts. Any HF was defined as a composite outcome including HFpEF, HFrEF, and HF with unknown ejection fraction (HFuEF). Effect estimates from both cohorts were meta-analyzed using fixed effects. Follow-up in the WHI was truncated at 22 years due to deviations from proportional hazards.

© 2024 Schuermans A et al. *JAMA Network Open.*

eTable 7. Multivariable-adjusted associations of clonal hematopoiesis of indeterminate potential (CHIP) and gene-specific CHIP subtypes with variant allele fraction (VAF) >10% with incident HF with preserved and reduced ejection fraction (HFpEF and HFrEF).

	**Jackson Heart Study**			**Women's Health Initiative**			**Meta-analysis**			
	**Cases / total *n***	**HR (95%CI)**	***P*-value**	**Cases / total *n***	**HR (95%CI)**	***P*-value**	**HR (95%CI)**	***P*-value**	***P*-value for between-study heterogeneity (Cochran *Q*)**	***I*² statistic (%)**
**Any HF**	-	-	-	-	-	-	-	-	-	-
Any CHIP	16 / 102	1.09 (0.65 to 1.83)	0.74	55 / 335	1.21 (0.91 to 1.59)	0.19	1.18 (0.92 to 1.51)	0.19	0.74	0.0
DNMT3A CHIP	10 / 63	1.08 (0.57 to 2.06)	0.81	28 / 201	0.98 (0.67 to 1.43)	0.90	1.00 (0.72 to 1.39)	0.99	0.79	0.0
TET2 CHIP	6 / 20	3.36 (1.47 to 7.68)	0.004	17 / 77	1.93 (1.19 to 3.14)	0.008	2.23 (1.46 to 3.39)	<0.001	0.26	22.0
**HFpEF**	-	-	-	-	-	-	-	-	-	-
Any CHIP	7 / 102	0.98 (0.45 to 2.13)	0.96	30 / 335	1.32 (0.91 to 1.93)	0.15	1.25 (0.89 to 1.76)	0.20	0.59	0.0
DNMT3A CHIP	3 / 63	0.65 (0.20 to 2.09)	0.47	18 / 201	1.24 (0.77 to 2)	0.39	1.13 (0.72 to 1.76)	0.60	0.32	0.0
TET2 CHIP	3 / 20	4.04 (1.24 to 13.14)	0.02	8 / 77	1.95 (0.96 to 3.96)	0.07	2.36 (1.29 to 4.35)	0.006	0.30	7.1
**HFrEF**	-	-	-	-	-	-	-	-	-	-
Any CHIP	4 / 102	0.66 (0.24 to 1.82)	0.42	12 / 335	0.78 (0.44 to 1.41)	0.42	0.75 (0.45 to 1.25)	0.27	0.77	0.0
DNMT3A CHIP	2 / 63	0.52 (0.13 to 2.15)	0.37	3 / 201	0.32 (0.1 to 1.01)	0.05	0.39 (0.16 to 0.95)	0.04	0.60	0.0
TET2 CHIP	2 / 20	2.38 (0.58 to 9.83)	0.23	4 / 77	1.29 (0.48 to 3.5)	0.61	1.58 (0.70 to 3.57)	0.27	0.49	0.0

Hazard ratios (HRs) with corresponding 95% confidence intervals (CIs) were calculated using Cox proportional hazards models adjusted for age at blood draw, income, education, smoking, body mass index, systolic blood draw, antihypertensive medication use, prevalent diabetes mellitus, coronary artery disease, and stroke. JHS models were further adjusted for sex, while WHI models were further adjusted for race/ethnicity and

© 2024 Schuermans A et al. *JAMA Network Open.*

inverse probability weights for inclusion in the TOPMed and HF adjudication cohorts. Any HF was defined as a composite outcome including HFpEF, HFrEF, and HF with unknown ejection fraction (HFuEF). Effect estimates from both cohorts were meta-analyzed using fixed effects. Follow-up in the WHI was truncated at 22 years due to deviations from proportional hazards. Follow-up occurred over a median of 12.0 (IQR: 11.0-12.0) years in the JHS and 15.3 (IQR: 9.0-22.0) years in the WHI.

© 2024 Schuermans A et al. *JAMA Network Open.*

eTable 8. Multivariable-adjusted associaitons of clonal hematopoiesis of indeterminate potential (CHIP) with HF with preserved and reduced ejection fraction (HFpEF and HFrEF) by C-reactive protein (CRP) <2 vs. ≥2 mg/L.

	**Women's Health Initiative**		
	**Cases / total *n***	**HR (95% CI)**	***P*-value**
**Any HF**	-	-	-
No CHIP and CRP <2 mg/L	150 / 1,334	Ref.	Ref.
No CHIP and CRP ≥2 mg/L	326 / 2,132	1.31 (1.06 to 1.61)	0.01
CHIP and CRP <2 mg/L	17 / 110	1.41 (0.85 to 2.34)	0.18
CHIP and CRP ≥2 mg/L	40 / 186	1.86 (1.29 to 2.66)	<0.001
**HFpEF**	-	-	-
No CHIP and CRP <2 mg/L	77 / 1,334	Ref.	Ref.
No CHIP and CRP ≥2 mg/L	153 / 2,132	1.17 (0.87 to 1.57)	0.29
CHIP and CRP <2 mg/L	9 / 110	1.43 (0.71 to 2.86)	0.32
CHIP and CRP ≥2 mg/L	23 / 186	1.94 (1.20 to 3.15)	0.007
**HFrEF**	-	-	-
No CHIP and CRP <2 mg/L	52 / 1,334	Ref.	Ref.
No CHIP and CRP ≥2 mg/L	117 / 2,132	1.52 (1.07 to 2.16)	0.02
CHIP and CRP <2 mg/L	6 / 110	1.50 (0.64 to 3.52)	0.35
CHIP and CRP ≥2 mg/L	6 / 186	0.98 (0.42 to 2.31)	0.96

Hazard ratios (HRs) with corresponding 95% confidence intervals (CIs) were calculated using Cox proportional hazards models adjusted for age at blood draw, race/ethnicity, income, education, smoking, body mass index, systolic blood draw, antihypertensive medication use, prevalent diabetes mellitus, coronary artery disease, stroke, CRP measurement assay, and inverse probability weights for inclusion in the TOPMed and HF adjudication cohorts. Any HF was defined as a composite outcome including HFpEF, HFrEF, and HF with unknown ejection fraction (HFuEF). Effect estimates from both cohorts were meta-analyzed using fixed effects. Follow-up was truncated at 22 years due to deviations from proportional hazards. Follow-up occurred over a median (IQR) of 15.3 (9.0-22.0) years.

© 2024 Schuermans A et al. *JAMA Network Open.*

eReferences

1. Keku, E. *et al.* Cardiovascular disease event classification in the Jackson Heart Study: methods and procedures. *Ethn Dis* **15**, S6-62 (2005).
2. Hansen, A. L. *et al.* Adverse Pregnancy Outcomes and Incident Heart Failure in the Women's Health Initiative. *JAMA Netw Open* **4**, e2138071–e2138071 (2021).
3. Jiang, Y., Scott, A. J. & Wild, C. J. Secondary analysis of case-control data. *Stat Med* **25**, 1323–1339 (2006).

© 2024 Schuermans A et al. *JAMA Network Open.*
