# Supplementary material for: Clonal Hematopoiesis and Incident Heart Failure With Preserved Ejection Fraction
Source: JAMA Netw Open. 2024 Jan 25;7(1):e2353244. doi: 10.1001/jamanetworkopen.2023.53244 (PMC10811556; doi:10.1001/jamanetworkopen.2023.53244)
Supplement: Supplement 2. — Data Sharing Statement [file jamanetwopen-e2353244-s002.pdf]

Data Sharing Statement

Schuermans. Clonal Hematopoiesis and Incident Heart Failure With Preserved Ejection Fraction. *JAMA Netw Open*. Published January 25, 2024.
doi:10.1001/jamanetworkopen.2023.53244

Data
Data available: No

Additional Information
Explanation for why data not available: Individual whole-genome sequence data for TOPMed whole genomes and individual-level phenotypes are available through restricted access via dbGaP.
